# The Vasopressin 1a Receptor Antagonist SRX246 Reduces Aggressive Behavior in Huntington’s Disease

**DOI:** 10.3390/jpm12101561

**Published:** 2022-09-22

**Authors:** Hilda T. Maibach, Michael J. Brownstein, Steven M. Hersch, Karen E. Anderson, Debra E. Itzkowitz, Eve M. Damiano, Neal G. Simon

**Affiliations:** 1 Azevan Pharmaceuticals Inc., Bethlehem, PA 18015, USA; 2 Eisai Pharmaceuticals, Nutley, NJ 07110, USA; 3 Formerly at Department of Neurology, Massachusetts General Hospital, Boston, MA 02114, USA; 4 Department of Psychiatry and Department of Neurology, Medstar Georgetown University Hospital, Washington, DC 20007, USA; 5 Department of Biological Sciences, Lehigh University, Bethlehem, PA 18015, USA

**Keywords:** Huntington’s disease, vasopressin 1a receptor antagonist, irritability, aggression

## Abstract

SRX246, an orally available CNS penetrant vasopressin (VP) V1a receptor antagonist, was studied in Huntington’s disease (HD) patients with irritability and aggressive behavior in the exploratory phase 2 trial, Safety, Tolerability, and Activity of SRX246 in Irritable HD patients (STAIR). This was a dose-escalation study; subjects received final doses of 120 mg BID, 160 mg BID, or placebo. The compound was safe and well tolerated. In this paper, we summarize the results of exploratory analyses of measures of problematic behaviors, including the Cohen–Mansfield Agitation Inventory (CMAI), Aberrant Behavior Checklist (ABC), Problem Behaviors Assessment-short form (PBA-s), Irritability Scale (IS), Clinical Global Impression (CGI), HD Quality of Life (QoL), and Caregiver Burden questionnaires. In addition to these, we asked subjects and caregivers to record answers to short questions about mood, irritability, and aggressive conduct in an eDiary. STAIR was the first rigorously designed study of behavioral endpoints like these in HD. The exploratory analyses showed that SRX246 reduced aggressive acts. Readily observed behaviors should be used as trial endpoints.

## 1. Introduction

Irritability and aggressive behavior are among the first symptoms of Huntington’s disease [1]. These problems have been reported in 40–70% of people with HD and are highly distressing for patients and their family members [2,3]. The behaviors are among the leading causes of institutionalization, but their presence often makes placement and retention in institutions difficult. Aggressive behavior is especially troublesome because of the physical risks to patients, family members, and caregivers. It is currently treated off-label with a number of different drugs, notably antipsychotics, antidepressants, anxiolytics, and mood stabilizers [4,5,6]. These are not very effective, and they have significant side effects. There is a significant unmet need for new, safer drugs that can manage these symptoms in HD patients as well as people with other neurodegenerative diseases.

SRX246 targets the V1a receptor, the most abundant VP receptor subtype in the brain [7,8]. This receptor is found throughout the cortex and limbic system and plays a prominent role in circuits known to modulate socioemotional responses to stimuli that elicit aggression/fear [9,10,11,12]. In preclinical studies, SRX246 demonstrated efficacy in models of aggression, depression, anxiety, and fear, and in a trial in healthy human volunteers, it significantly attenuated the effect of intranasal VP, which increases BOLD signals in circuits mediating fear [10,12]. The compound is orally available, CNS penetrant, and highly specific for the V1a receptor [13,14]. The drug candidate had an excellent safety profile in animals, and based on clinical studies, including the STAIR study (NCT 02507284), it was safe and well-tolerated in human subjects as well [15].

STAIR was designed to assess the tolerability and safety of SRX246 in HD patients with irritability and aggressive behavior. Behavioral assessments were exploratory. We tested scales including the CMAI and ABC that had been used in individuals with other neurodegenerative diseases and an electronic diary that focused on mood and aggressive behavior. Other scales that we employed have been used in HD patients: the PBA-s, IS, HD QoL, and Caregiver Burden. Our goal was to determine if SRX246 would reduce irritability and aggression in HD patients and identify an outcome measure for aggressive behavior in this population. The results were encouraging and led to the FDA granting an orphan designation and fast-track status for the use of SRX246 in the treatment of HD.

## 2. Materials and Methods

### 2.1. Study Design

This was a 3-arm, multicenter, randomized, placebo-controlled, double-blind, 12-week, dose-escalation study. The subjects, who were recruited in 22 NINDS-supported NeuroNext centers in the US, were required to have a study partner to assist and answer questions about them. Both the patient and study partner provided written informed consent. The subjects had to be 18 years old or older and were required to have motor, cognitive, or behavioral features of HD, a confirmatory family history of HD or a CAG repeat expansion > 36, a Total Functional Capacity (TFC) score on the UHDRS of 5–13, and evidence of irritability and/or aggression—specifically, a severity score of 2 (=Mild) or more for the UHDRS Irritability question, 30b or Aggression question, 31b. Women of childbearing potential had to have a negative pregnancy test and must have been using adequate contraception during the study. Men must have agreed to use contraception. Subjects had to be able to swallow the drug capsules and have sufficient English skills to complete all required assessments without the assistance of an interpreter. Subjects could not have severe psychiatric symptoms, systemic illnesses, or disabilities that could lead to difficulty complying with the protocol; a history of alcohol or substance abuse in the 2 years preceding the study; or active suicidal ideation. Patients who were taking prescription or over-the-counter medications could continue to do so. Ninety-four percent of subjects used such medications; 50% of the participants took antidepressants, and 35% took antipsychotics.

Patients were randomly assigned to one of three treatment arms: 120 mg SRX246, 160 mg SRX246 or placebo. All capsules provided were identical in appearance, and subjects took one capsule twice a day (BID) for a total of 12 weeks.

In the first 2 weeks, subjects in the active arms were given 80 mg of SRX246 twice daily (BID). This dose was included as a safety step because the HD patients were frequently on concomitant medications and more fragile than other people who have been exposed to SRX246 (e.g., NCT02055638, NCT02922166). It was not expected to be efficacious. Patients were then given 120 mg of drug BID for 4 weeks. At visit 4 (week 6), they either continued on 120 mg or received 160 mg BID for an additional 6 weeks.

Clinical assessments were conducted at baseline, visit 4 (week 6) and visit 7 (week 12) (Supplementary Materials, Table S1). Patients who could not tolerate their dose of the drug (or placebo) could have their dose reduced. While the investigators were blind to treatment, they could decide to decrease the dose of the drug (or placebo). The change was effected by the local pharmacists. Temporary suspension and/or complete drug discontinuation was completed in a manner that protected the study blind as well. Patients who discontinued the study drug were encouraged to stay on protocol and complete study visits.

The study was approved by a central Institutional Review Board (IRB; Massachusetts General Hospital) as part of the NeuroNext program and secondarily by IRBs at the 22 participating sites. It was registered at www.clinicaltrials.gov, identifier NCT2507284 [16]. The roles of the site’s principal investigators (PIs), PI, IRB, Independent Medical Monitor, and members of the Data Safety Monitoring Board (DSMB) in guaranteeing the safety of trial participants are described in an NINDS Guidelines document (16). There were no explicit stopping rules in the protocol.

### 2.2. Assessments

In seeking evidence of drug activity, we looked for verbal or physical outbursts. Problem behaviors were based on eDiary reports by patients and study partners and scales administered during clinical visits: the Unified Huntington’s Disease Rating Scale (UHDRS), Aberrant Behavior Checklist-I (ABC-I), Problem Behavior Assessment-short form (PBA-s), Irritability Scale (IS), Caregiver Burden Assessment (CGB), HD Quality of Life, and Cohen Mansfield Agitation Inventory (CMAI). A full schedule of visits and information about the scoring of the scales used are included in the Supplemental Material.

The UHDRS is the most commonly used assessment of HD. It is divided into four domains: motor performance (TMS), cognitive function (Cognitive), behavioral abnormalities (including Irritability and Aggression), Independence, and functional capacity (TFC). TFC is the most frequently used measure of function in HD. It lists disease burden by stage (ranging from 1 to 5). Patients in stage 1 have the least evident and mild disease; stage 3 patients need assistance; stage 5 patients are severely affected.

The PBA-s [17] is a semi-structured interview in which the investigator ascertains the frequency and severity of neuropsychiatric symptoms by observation and reports of symptoms by subjects and study partners.

The IS is a self-administered questionnaire that collects information about different aspects of irritability [18,19]. The IS was completed separately by subjects and study partners.

The ABC-I, one of 5 established domains of the ABC-C [20], targets behaviors associated with irritable agitation (temper tantrums) and mood (depression, mood lability) in children or adults with cognitive or developmental delays.

The CMAI [21] was developed to record behaviors in older adults in nursing homes. Study partners are asked to rate the frequency of physically aggressive, physically non-aggressive, and verbally agitated behaviors [22].

The HD Quality of life (QoL) scale was scored by subjects with input from the study partner if necessary. In our analysis, we used exploratory factor analysis to identify subscores.

The Caregiver Burden Score (CGB) was adapted from the Caregiver Burden Inventory instrument [23]. It has been validated for the HD population relative to TFC [24].

Finally, we used an electronic diary (ePRO). Our ePRO instrument, which was completed on a near-daily basis by patients and twice weekly by study partners, asked about physical (hitting) and verbal (yelling) aggression and rated the intensity of moods (feeling sad, stressed, angry, impatient, or irritated).

### 2.3. Statistical Methods

In doing exploratory analyses, we looked at the activity of SRX246 on poor control of temper leading to verbal or behavioral outbursts. By doing this, we hoped to identify ways to measure these negative behaviors. We examined total scores, subscores and individual item scores from a wide range of scales: quality of life (patient-reported), care burden (study partner-reported), moods such as anger or irritability (study partner-reported), and aggressive behaviors (study partner-reported) as well as patient characteristics and disease symptoms. We first analyzed baseline data to learn the relationship between patient characteristics, disease symptoms, and scale measurements prior to any intervention.

Statistical analysis of baseline data included summaries, assessments of data distributions and homogeneity tests. Simple correlations and general linear models were used to examine the relationship between demographics (e.g., gender), disease duration, total function, and endpoints, with a specific focus on those covariates that contribute to the heterogeneity of treatment effect. We incorporated what we learned from the baseline analyses into models that we used to evaluate treatment responses.

Treatment response was evaluated using both a linear mixed model (LMM) repeated measures method and an analysis of covariance (ANCOVA). The LMM estimated rates of change over time by treatment group. ANCOVA least squares (LS) means and Type III SS *p*-values were used to identify predictive variables and determine the best fit model (maximum R^2^, *p*-values, and least intercorrelation between covariates) with the fewest possible covariates.

To evaluate the impact of different assumptions or methods on the results, we conducted a series of sensitivity analyses, including study site differences [25] and response patterns by patient subgroup. The subgroups were (1) pre-specified modifiers: age, sex, education, UHDRS TFC Stage, CAP scores, and CGI-severity; and (2) posthoc variables: UHDRS aggressive behavior severity; evident aggression at baseline and reported history of violence. We looked at dose response and the consistency of trends with SRX 160 mg and 120 mg BID dose groups combined into a single active treatment group.

All analyses were conducted in SAS 9.4 and JMP 16.2.0 (Cary NC).

## 3. Results

Baseline: The primary and secondary endpoints of the STAIR study were the tolerability and safety of SRX246. These results have been reported earlier [15]. Between 2016 and 2018, 106 patients and their study partners were enrolled at 22 US study sites. The patients, whose average age was 50, ranged in age from 19 to 77 (Table 1). Fifty-two percent were female. Only 12 of the 106 were under 35 years of age; 8 of these were female. All patients except for a single “mixed” race individual described themselves as “white”. Six percent of the people enrolled were “Hispanic”. There was a full range of educational attainment; 50% of the patients had at least an associate degree (Supplementary Material Table S2). None of our analyses indicated education-related effects on our endpoints of interest.

The patients’ CAG repeat lengths ranged from 38 to 58, and all but 3 patients (all over the age of 65) had repeats greater than or equal to 40. The average age at diagnosis was 47, and the time from diagnosis to study enrollment ranged from 0 to 29 years. There were no differences in mean CAG repeat lengths by sex, and the distribution by sex did not vary with age.

Treatment groups, as randomized, were similar in race, ethnicity, sex, and education, but the patients in the 160 mg BID group were more independent (UHDRS) and had higher functional capacities (50% were in TFC Stage 1) than others. Members of the 160 mg group were also less anxious than placebo-treated patients (PBA *p* < 0.05), and few displayed aggressive behavior (Hitting on CMAI, 6% vs. 20% placebo). It is important to note that they had the lowest fraction of study partners from the same household (74% versus 92% placebo).

Study partners were most often family members (spouses or parents) who lived with the patient (82%). The patients’ TFC, CAG, and average age did not differ as a function of study partner household membership, but study partners who were household members reported higher caregiver burden scores than those who were not (21.8 vs. 13.5, respectively, *p* < 0.05), more frequent and severe anger in patients (PBA-s), and higher irritability scores (ABC-I and IS).

Patients were eligible for enrollment if they were at least “Mildly irritable” or “Mildly aggressive” (UHDRS questions 30b and 31b, respectively), but fewer than half of our subjects were physically aggressive regardless of gender (Table 2). Neither irritability nor aggression was significantly correlated with CGI-S scores, which rated the majority of patients as “normal”, “borderline”, or “mildly ill.”

Based on UHDRS disease stage classifications, aggressive behavior and other problematic symptoms were more prevalent as TFC declined (Table 3). For example, 55% of the patients had a reported medical history of violent behavior, and those in TFC Stage 3 were much more often violent (84%).

Overall, caregiver burden (CGB) scores were not high. At baseline, 82% of the CGB scores were “mild” (<40). The mean was 27.7 (+/−14.9). The UHDRS assessments of total functional capacity (TFC), total motor function (TMS), and independence were significantly correlated with caregiver burden scores. Caregiver burden increased as functional capacity declined. Study partners of patients who required assistance most of the day (TFC Stage 3) had the highest CGB scores.

HD quality of life (QoL) scores (as reported by patients) were strongly correlated with (UHDRS) independence scores, depressed mood, anxiety (PBA-s), ocular function, dystopia, TFC Stage, and irritability (PBA-s), R^2^ = 0.40, (*p* < 0.0001). However, the QoL scores were not correlated with aggressive behaviors (UHDRS, CMAI, or ePRO; see Supplementary Material Table S3 for correlations). TFC Stage 2 patients, on average, reported the worst quality of life.

**Figure 1:**
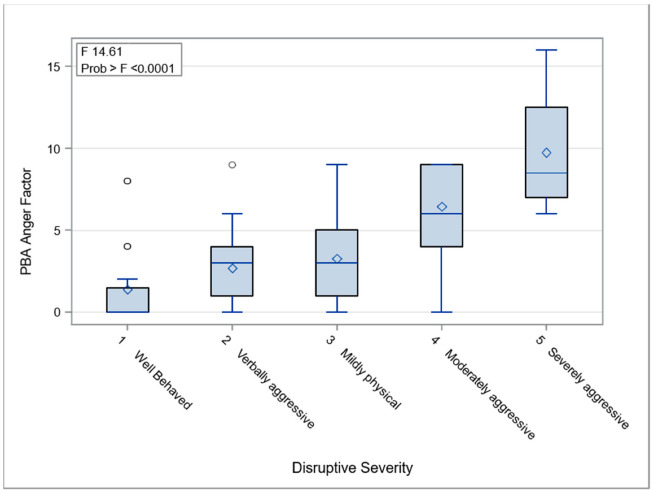
PBA Anger Factor Score by UHDRS Aggressive Behavior Severity at baseline.

The frequency and severity of PBA anger (as a product score) increased significantly with the severity of aggressive/disruptive behaviors (Figure 1). Patients with a history of violence were more likely to be aggressive/disruptive based on UHDRS (chi^2^, *p* < 0.0001, Table 4). This was also evident in our subjects’ ePRO data; anger and yelling were present 99% of the time when hitting was reported by either the patient or the study partner. Patients who were classified as “problematic” (ABC-I > 14) were three times as likely to be “physically aggressive” (CMAI, *p* < 0.0001), and those who were problematic or had a history of violence had more aggressive events (ePRO hitting or yelling, patient- or partner-reported), (*p* < 0.02).

Disease burden was strongly correlated with, and often the primary predictor of, increased caregiver burden, decreased quality of life, and irritability (IS and PBA-s). Irritability (partner reported) and caregiver burden were significantly lower when reported by study partners who were not members of the patient’s household. Based on ePRO data, many patients exhibited violent behavior infrequently and episodically. Aggression was correlated with anger but not irritability scores. For both ePRO and efficacy scale data, verbal outbursts strongly predicted the severity/frequency of aggression. We did not find significant differences in aggressive behaviors based on CGI-S, age, sex, or education.

*Treatment effects*: HD Quality of Life, caregiver burden, CMAI Total score and PBA-Anxiety factor mean scores did not change from baseline for any of the treatment groups. PBA Anger and Irritability factor scores, IS partner-reported, and ABC Total Score did. The changes were similar in the two (120 mg and 160 mg BID) treatment groups.

Our ePRO data provided more opportunities to capture information about aggressive episodes from patients and study partners. Our first evidence that SRX246 might reduce aggressive behavior was based on these reports. When we compared the proportion of patients at baseline who “hit someone” or “hit something” to the proportion at the end of the treatment period, we saw that violent behavior decreased in those given SRX246 but not placebo (*p* < 0.001, Table 5). Additional analyses in the subgroup of patients with a higher disease burden (CAP_warner_ > 100) indicated that the proportion of patients who experienced a reduction in the number of aggressive events (ePRO: hit or yell) was 65% in the SRX246 group compared to 22% in the placebo group, RR = 2.9; chi^2^ *p* < 0.03). Based on this finding, we returned to the scales described above and used.

Exploratory factor analyses (EFAs) were used to assess patterns within our efficacy scales. In the case of the CMAI, we wanted to know if aggression in HD patients was similar to, or different from, the populations for whom the questionnaires were originally intended—elderly dementia patients whose behaviors included aggression [28,29]. The analyses with either 3- or 4-factor solutions proved useful. With three factors (non-physical aggression, agitation, and aggressive behaviors), the results matched those originally described by Cohen-Mansfield (1986) [30]. Our four-factor solution distinguished between physical/verbal aggressive behaviors and physical/verbal non-aggressive behaviors. This solution mirrored work in Alzheimer’s disease [31,32]. We derived our composite endpoint for aggression based on these results. A similar analysis of the ABC-I scale indicated that the severity of angry outbursts and temper tantrums were also useful measures of changes in aggression. Using informative CMAI and ABC-I terms as endpoints, our analyses supported the hypotheses that physical aggression and/or the inability to control responses to perceived threats were associated with disease stage [33], more severe anger (PBA Scores), and verbal aggression, *p* < 0.0001.

The TFC and PBA anger scores, treatment group, and week of visit explained a large proportion of the variability in our endpoints (R^2^ ranged from 0.36 to 0.59). Distinct behaviors such as hitting (SRX246 120 mg difference, *p* < 0.0002, Table 6) or a combination of aggressive behaviors—i.e., the CMAI aggression subscore—decreased more in SRX246-treated patients than in those who received placebo by the end of the treatment period.

We used sensitivity analyses to understand the marked improvements from baseline seen in both the placebo and SRX246 groups for endpoints such as the PBA-Irritability Factor and the ABC Total scores. The placebo effect was most apparent in patients with mild disease (TFC Stage 1). Excluding patients in TFC Stage 1 improved the precision of the estimates and increased the difference between SRX246 and placebo. Sensitivity analyses indicated that aggressive behaviors measured by ePRO, UHDRS, and CMAI scores were, on average, higher in patients with histories of violence and drug-induced differences in aggression were most evident in the subset of TFC Stage 2 and 3 patients with such histories.

## 4. Discussion

Many HD patients are irritable and aggressive [2,3]. These behaviors can be highly distressing for patients, family members, and caregivers. They negatively impact quality of life and are among the leading causes of institutionalization in the HD population. Currently, aggression is treated off-label with antipsychotics, antidepressants, anxiolytics, and mood stabilizers [4,5,6]. Unfortunately, these are not very effective and have significant side effects. We need safer, more effective drugs to control aggressive behaviors in HD patients and individuals with other neurodegenerative diseases.

In the CNS, vasopressin increases aggressive behavior in multiple species, including humans [9,10]. The V1a receptor, the dominant vasopressin receptor subtype in the brain, is the target for SRX246. This receptor modulates the response to negative socioemotional stimuli via actions in fear circuits [10,12,34]. In patients with Intermittent Explosive Disorder, SRX246 decreased the number of severe aggressive episodes and reduced lost work and school days, NCT02055638 [35]. Based on this and other clinical observations, the STAIR trial was undertaken with two main goals. One was to test the safety and tolerability of SRX246, a highly selective, highly specific V1a receptor antagonist, in HD patients. We found the drug was well-tolerated and safe [1]. SRX246 did not increase apathy, measured on the PBA-s Apathy item, which is a bothersome side effect of many off-label drugs currently used to treat aggressive behaviors in HD. Our other main objective was to assess the drug as a treatment for aggressive behaviors and to identify a scale to use in measuring these behaviors in the HD population. This work was exploratory because similar studies had not been undertaken in the past.

Our results showed that SRX246 reduced aggressive behavior in selected members of the per-protocol patient population. More specifically, those who were “responders” typically were in Stage 2 or Stage 3 of the disease and had a history of violence. Analyses of ePRO data in this patient subgroup revealed a significant decrease in “hitting” and “yelling” in SRX246-treated patients compared to placebo-treated patients by the end of the treatment period. This finding was supported by differences in CMAI and ABC reported behaviors in analyses that considered the patients’ TFC stage, PBA-anger score, and medical history of violence.

Our results indicate the detection of changes in aggression must be based on frequent reporting of objective, well-defined, and clearly understood measures of episodic behaviors. Aggression may take different forms in different individuals—hitting in some cases, pushing, kicking, and/or intense verbal outbursts in others. To measure the disturbance in any given patient, a scale is needed that includes several different aggressive acts. We found that the most reliable way to measure aggression was to frequently ask caregivers and patients about objective acts of violence (e.g., hitting, yelling) that are unambiguous and easily understood. These types of objective measures (e.g., ePRO hitting, CMAI hitting, and ABC outbursts and temper tantrums) correlate well with one another. We did not find treatment-based benefits in measures of mood, including irritability. While this finding may seem surprising in light of our observation that patients who hit are highly irritable, perhaps it should not be because irritability and aggression, although strongly related, are not the same. Our patients were reportedly irritable almost every day, but only about 50% were physically aggressive during the course of the study. Calling a patient irritable requires that one make a judgement about his or her internal state, which makes assessing irritability difficult. Our main irritability measure, the IS, has only been studied cross-sectionally; there are no data on its ability to show changes over time or in a clinical trial setting [18,19,36].

Aggressive behavior is highly problematic for HD patients and their caregivers. It is a leading cause of institutionalization, and it negatively impacts social interactions. Safe, effective drugs that can decrease violent behavior without significant side effects are needed. SRX246 is a promising candidate. It appears to have fewer side effects than drugs used off-label to treat aggression in HD, and we have provided evidence that it may be efficacious for a significant number of HD patients, specifically Stage 2 and Stage 3 individuals with a history of violence. Our phase 3 studies will incorporate the information that emerged from the exploratory analyses of the STAIR trial. We look forward to seeing a clinically meaningful change in HD patients who suffer from aggressive behavior.

## Figures and Tables

**Table 1:** Patient characteristics at baseline, (n = 106).

	Mean	Standard Deviation	Median	Min	Max	10th%	90th%
Age	50.0	12.0	51.0	19.0	77.0	34.0	64.0
Age at diagnosis	47.0	12.0	48.0	19.0	77.0	31.0	61.0
CAG	44.0	4.0	43.0	38.0	58.0	40.0	48.0
CAP ^1^	463.0	105.0	463.0	180.0	737.0	329.0	604.0
CAP ^2^	100.0	18.0	100.0	41.0	140.0	74.0	121.0
Disease Duration (yrs.)	5.0	4.0	4.0	0.0	29.0	1.0	11.0
UHDRS Independence	82.0	13.0	80.0	50.0	100.0	70.0	100.0
Total Functional Capacity (TFC)	9.0	3.0	10.0	5.0	13.0	6.0	12.0
Total Motor Score (TMS)	27.3	16.2	26.0	1.0	85.0	5.5	49.0
Total Dystonia Score	1.8	2.7	1.0	0.0	13.0	0.0	5.0
Total Chorea Score	7.8	4.9	7.0	0.0	21.0	1.0	14.0
Dysarthria	0.5	0.6	0.0	0.0	3.0	0.0	1.0
Bradykinesia Body	0.9	0.9	1.0	0.0	3.0	0.0	2.0
Gait	0.8	0.6	1.0	0.0	2.0	0.0	2.0
Tandem Walking	1.4	1.1	1.0	0.0	4.0	0.0	3.0
Retropulsion Pull Test	0.4	0.7	0.0	0.0	3.0	0.0	2.0
Luria	1.1	1.2	1.0	0.0	4.0	0.0	3.0
Stroop Color Naming	51.0	17.0	49.0	21.0	97.0	31.0	74.0
SDMT	32.0	14.0	31.0	9.0	76.0	16.0	51.0
Verbal Fluency F-A-S	28.0	14.0	27.0	3.0	81.0	10.0	43.0

^1,2^ see [26,27] for CAP score derivation.

**Table 2:** The number of patients classified by severity of UHDRS Irritability and Aggressive/Disruptive Behaviors.

		Aggressive/Disruptive Severity						
		None	Verbal Only	Mildly Physical	Moderate	Severe	Total	
**Irritability Severity**	None	0	0	0	0	0	0	0%
	Slight	0	0	2	0	0	2	2%
	Mild	14	16	7	0	0	37	35%
	Moderate	5	22	18	15	4	64	61%
	Severe	1	0	1	1	0	3	3%
	Total	20	38	28	16	4	106	
		19%	36%	27%	15%	4%		

**Table 3:** Medical History of Psychiatric Symptoms by UHDRS TFC Stage.

		TFC Stage		All
	1	2	3	
	(11–13)	(7–10)	(5–6)	
	n = 33	n = 54	n = 19	N = 106
Irritability	33 (100%)	54 (100%)	19 (100%)	106 (100%)
Anxiety	25 (76%)	44 (82%)	16 (84%)	85 (80%)
Apathy	20 (61%)	37 (69%)	9 (48%)	66 (62%)
Cognitive dysfunction	13 (39%)	35 (65%)	14 (74%)	62 (59%)
Depression	25 (76%)	46 (85%)	13 (68%)	84 (79%)
Obsessive	16 (49%)	27 (50 %)	14 (74%)	47 (44%)
Psychosis	3 (9%)	4 (7%)	3 (16%)	10 (9%)
Psychiatric Hospitalization	2 (6%)	4 (7%)	7 (37%)	13 (12%)
HX Violence	18 (55%)	25 (46%)	16 (84%)	59 (56%)

**Table 4:** The number of patients classified on Severity of Aggressive/Disruptive behaviors by medical history of violence based on UHDRS question 31.

Aggressive/Disruptive Severity						
History of Violence	None	Verbal Only	Mild	Moderately	Severely	N
No	17	20	5	3	1	46
Yes	3	18	22	12	3	58

**Table 5:** ePRO estimate of the number of patients who “hit” at any time based on either patient or study partner reported incidence.

		Baseline	End of Treatment
Placebo	n (%)	8 (24%)	10 (33%)
	Total	34	30
SRX246	n (%)	29 (53%)	20 (37%)
	Total	54	54

**Table 6:** Study Partner Reported Hitting, CMAI Scale, and LS means by Treatment and Visit, Per Protocol Population.

Visit	Treatment Group	Hitting LSMEAN
2	80 + 120 + 120	1.72
	80 + 120 + 160	1.02
	Placebo	1.13
7	80 + 120 + 120	1.06
	80 + 120 + 160	1.07
	Placebo	1.25

## Data Availability

The data presented in this study are openly available at AVN011.indigordd-share.com.

## Supplementary Materials

The following supporting information can be downloaded at: https://www.mdpi.com/article/10.3390/jpm12101561/s1, Table S1: Schedule of Visits and Assessments; Table S2: Baseline Demographics with Education and CGI-Severity; Table S3: Correlations.
