# Supplementary material for: The Vasopressin 1a Receptor Antagonist SRX246 Reduces Aggressive Behavior in Huntington’s Disease
Source: J Pers Med. 2022 Sep 22;12(10):1561. doi: 10.3390/jpm12101561 (PMC9605366; doi:10.3390/jpm12101561)
Supplement: Supplementary file 1 [file jpm-12-01561-s001.zip › jpm-1820923-supplementary.pdf]

Supplementary Table S1. The schedule of visits and assessments conducted from screening to the end of study visit. In addition, participants and their study partners (caregivers) answered questions in an eDiary (electronic Patient Reported Outcome; ePRO) about mood and aggressive outbursts using a web application that was developed for this study.

Week	SC	BL	2	4	6	8	10	12	F/U
Visit Window	Day -30 to 0	Day 0	Day 14±5	Day 28±5	Day 42±5	Day 56±5	Day 70±5	Day 84±5	Day 91±5
Visit	SC	BL	01	T1	02	03	04	05	T2
**Assessment** / **Dosing (BID)**	**none**	**80 mg**	**120 mg**		**120 or 160mg**				**none**
Review Study Drug Compliance			X	X	X	X	X	X	
Assessments for Adverse Events		X	X	X	X	X	X	X	X
Assessments for ConcomitantMedicationUse		X	X	X	X	X	X	X	X
Columbia Suicide Severity Rating Scale (CSSRS)	X	X	X		X	X	X	X	
Unified Huntington Disease Rating Scale (UHDRS)	X	X			X			X	
Clinical Global Impression-Severity of Illness(CGI-S)		X							
Clinical Global Impression-Improvement(CGI-I)					X			X	
Provide Diary and Instructions		X	R	R	R	R	R	R	
Aberrant Behavior Checklist (ABC), irritability subscale		X			X			X	
Cohen-Mansfield Aggression Inventory(CMAI)		X			X			X	
ProblemBehaviorsAssessment-short form(PBA-s)		X			X			X	
Irritability Scale		X			X			X	
HD QoL		X			X			X	
Caregiver Burden Questionnaire		X			X			X	
Participant/StudyPartner/PI Blindedness Questionnaire								X	
Informed Consent (Subject)	X	R							
Informed Consent (Study Partner)	X	R[a]	R[e]	R[e]	R[e]	R[e]	R[e]	R[e]	
Assign Subject ID/ Unique Site ID number	X								
Inclusion/Exclusion Criteria	X	R							
Screening Demographics (Subject)	X								
Screening Demographics (Study Partner)	X	R[a]	R[e]	R[e]	R[e]	R[e]	R[e]	R[e]	
Medical & Surgical Historv	X	R							
Review methods of birth control with men and women	X	X							
Complete Phvsical Exam, including neurological	X							X	
Height, Weight (wt)	X				X(wt)			X(wt)	
Orthostatic Vital Signs (HR, BP), and Body Temperature	X	X[b]	X		X	X[b]	X	X	
Resting 12-Lead EKG	X	X	X		X	X	X	X	
Safety Labs (Hematology, Chemistry, Urinalysis, Study Drug Spot Check)	X	X[h]	X[g]		X[g]	X	X	X[g]	
Pregnancy Test (females only)	X(s)	X(u)			X (s)			X(s)	
Urine Drug Screen	X								
Blood Collections for CAGn Genotyping and storage		X	R[f]		R[f]	R[f]	R[f]	R[f]	
Randomization Assignment		X							
Dispense Study drug		X	X		X	X	X		
Instruct Subject and Study Partner about Study Dmg		X	X		X	X	X	X[c]	
Administer Study Drug on Site		X				X	(X)[d]		

a – must be completed by the site Investigator.
b – if not performed at screening.
c – conduct pre-dosing and 1 hour post first dose of administering study medication.
d – return all study drug supplies to clinic, do not issue additional study drug
e – to be completed by Subject and Informant
* – if not performed during Week 8, perform at Week 10
R – review; (s) – serum test; (u) – urine test
Definitions for Study Weeks/Visits: SC – Screening; BL – Baseline; T – Telephone Contact; F/U – Follow-up

Supplementary Table S2. Baseline Demographics with Education and CGI-Severity

Gender and Race n (%)		Education, n (%)		CGI-Severity n (%)	
Male	51 (48%)	High school diploma/GED or less	45 (42%)	Normal	9 (8.5%)
Female	55 (51.9%)	Technical or associate's degree/certificate	20 (19%)	Borderline	14 (13.2%)
		Bachelor's degree	26 (25%)	Mildly ill	43 (40.6%)
Race, white	105 (99%)	Master's degree	11 (10%)	Moderately ill	15 (14.2%)
Race, mixed	1 (1%)	Doctoral degree	4 (4%)	Severely ill	3 (2.8%)

Supplementary Table S3. Correlations

Variable	by Variable	Correlation	Signif Prob
Age	HDQol Total	-0.0125	0.90
ABC Total	Age	-0.1382	0.16
ABC Total	CAP	-0.1117	0.26
ABC Total	CMAI total	0.567	<.0001
ABC Total	DisruptiveFreq	0.367	0.0001
ABC Total	DisruptiveSev	0.476	<.0001
ABC Total	HDQol Total	0.148	0.14
ABC Total	Hitting	0.187	0.06
ABC Total	Independence	-0.0790	0.42
ABC Total	IS_partner	0.571	<.0001
ABC Total	PBA Angry Factor	0.482	<.0001
ABC Total	PBA Anxiety Factor	0.207	0.0340
ABC Total	PBA Irritabilty Factor	0.354	0.0002
CAP	Age	0.092	0.35
CAP	HDQol Total	0.024	0.81
CMAI total	Age	-0.0257	0.79
CMAI total	CAP	0.107	0.28
CMAI total	HDQol Total	0.128	0.20
DisruptiveFreq	Age	-0.0930	0.34
DisruptiveFreq	CAP	-0.0453	0.64
DisruptiveFreq	CMAI total	0.297	0.0021
DisruptiveFreq	DisruptiveSev	0.649	<.0001
DisruptiveFreq	HDQol Total	0.137	0.17
DisruptiveSev	Age	-0.2530	0.0089
DisruptiveSev	CAP	-0.0800	0.42
DisruptiveSev	CMAI total	0.303	0.0017
DisruptiveSev	HDQol Total	0.085	0.39
Hitting	Age	-0.0836	0.40

Hitting	CAP	0.120	0.22
Hitting	CMAI total	0.346	0.0003
Hitting	DisruptiveFreq	0.226	0.0205
Hitting	DisruptiveSev	0.396	<.0001
Hitting	HDQol Total	-0.0938	0.35
Hitting	Independence	-0.2630	0.0067
Independence	Age	-0.1691	0.08
Independence	CAP	-0.4674	<.0001
Independence	CMAI total	-0.2738	0.0047
Independence	DisruptiveFreq	-0.1700	0.08
Independence	DisruptiveSev	-0.1337	0.17
Independence	HDQol Total	-0.2406	0.0139
IS - partner	Age	-0.0525	0.60
IS - partner	CAP	-0.1114	0.26
IS - partner	CMAI total	0.360	0.0002
IS - partner	DisruptiveFreq	0.219	0.0256
IS - partner	DisruptiveSev	0.339	0.0004
IS - partner	HDQol Total	-0.0975	0.33
IS - partner	Hitting	0.204	0.0381
IS - partner	Independence	-0.0098	0.92
PBA Angry factor	Age	-0.1558	0.11
PBA Angry Factor	CAP	-0.1452	0.14
PBA Angry Factor	CMAI total	0.294	0.0023
PBA Angry Factor	DisruptiveFreq	0.442	<.0001
PBA Angry Factor	DisruptiveSev	0.567	<.0001
PBA Angry Factor	HDQol Total	0.103	0.30
PBA Angry Factor	Hitting	0.191	0.05
PBA Angry Factor	Independence	-0.0432	0.66
PBA Angry Factor	IS_partner	0.272	0.0052
PBA Anxiety Factor	Age	0.029	0.77
PBA Anxiety Factor	CAP	-0.1835	0.06

PBA Anxiety Factor	CMAI total	0.199	0.0419
PBA Anxiety Factor	DisruptiveFreq	0.099	0.32
PBA Anxiety Factor	DisruptiveSev	0.002	0.99
PBA Anxiety Factor	HDQol Total	0.302	0.0019
PBA Anxiety Factor	Hitting	-0.0320	0.75
PBA Anxiety Factor	Independence	0.084	0.40
PBA Anxiety Factor	IS_partner	0.201	0.0412
PBA Anxiety Factor	PBA Angry Factor	0.163	0.10
PBA Irritabilty Factor	Age	-0.0879	0.37
PBA Irritabilty Factor	CAP	-0.1430	0.15
PBA Irritabilty Factor	CMAI total	0.242	0.0128
PBA Irritabilty Factor	DisruptiveFreq	0.225	0.0208
PBA Irritabilty Factor	DisruptiveSev	0.300	0.0019
PBA Irritabilty Factor	HDQol Total	0.258	0.0086
PBA Irritabilty Factor	Hitting	0.119	0.23
PBA Irritabilty Factor	Independence	0.043	0.66
PBA Irritabilty Factor	IS_partner	0.269	0.0058
PBA Irritabilty Factor	PBA Angry Factor	0.581	<.0001
PBA Irritabilty Factor	PBA Anxiety Factor	0.425	<.0001
SDMT Score	ABC Total	-0.0281	0.78
SDMT Score	Age	-0.2090	0.0324
SDMT Score	CAP	-0.6096	<.0001
SDMT Score	CMAI total	-0.2228	0.0230
SDMT Score	DisruptiveFreq	-0.1332	0.18
SDMT Score	DisruptiveSev	-0.0640	0.52
SDMT Score	HDQol Total	-0.0743	0.46
SDMT Score	Hitting	-0.1055	0.29
SDMT Score	Independence	0.589	<.0001
SDMT Score	IS_partner	0.010	0.92
SDMT Score	PBA Angry Factor	0.053	0.59
SDMT Score	PBA Anxiety Factor	0.078	0.43

SDMT Score	PBA Irritabilty Factor	0.043	0.66
SDMT Score	Sex	0.142	0.15
SDMT Score	Verbal Fluency Test Score	0.659	<.0001
Sex	ABC Total	0.004	0.97
Sex	Age	-0.1396	0.15
Sex	CAP	-0.0069	0.94
Sex	CMAI total	-0.0388	0.69
Sex	DisruptiveFreq	0.027	0.78
Sex	DisruptiveSev	0.018	0.86
Sex	HDQol Total	0.126	0.20
Sex	Hitting	0.019	0.85
Sex	Independence	0.152	0.12
Sex	IS_partner	0.070	0.48
Sex	PBA Angry Factor	-0.0537	0.59
Sex	PBA Anxiety Factor	0.041	0.68
Sex	PBA Irritabilty Factor	0.109	0.27
Verbal Fluency Test Score	ABC Total	-0.0923	0.35
Verbal Fluency Test Score	Age	-0.0549	0.58
Verbal Fluency Test Score	CAP	-0.3985	<.0001
Verbal Fluency Test Score	CMAI total	-0.2051	0.0368
Verbal Fluency Test Score	DisruptiveFreq	-0.1582	0.11
Verbal Fluency Test Score	DisruptiveSev	-0.1190	0.23
Verbal Fluency Test Score	HDQol Total	-0.0323	0.75
Verbal Fluency Test Score	Hitting	-0.0652	0.51
Verbal Fluency Test Score	Independence	0.479	<.0001
Verbal Fluency Test Score	IS_partner	-0.0603	0.55
Verbal Fluency Test Score	PBA Angry Factor	-0.0294	0.77
Verbal Fluency Test Score	PBA Anxiety Factor	0.012	0.90
Verbal Fluency Test Score	PBA Irritabilty Factor	-0.0255	0.80
Verbal Fluency Test Score	Sex	0.054	0.58
